# Unravelling the synergistic potential of Olibanum extract and *Bacillus clausii* in combating intestinal cryptosporidiosis in immunosuppressed mice

**DOI:** 10.1371/journal.pntd.0014734

**Published:** 2026-09-30

**Authors:** Fadwa M. Arafa, Mohamed Hagar, Ahmed Zakaria, Eman Sheta, Nehal N. Hezema

**Affiliations:** 1 Department of Medical Parasitology, Faculty of Medicine, Alexandria University, Alexandria, Egypt; 2 Department of Chemistry, Faculty of Science, Alexandria University, Alexandria, Egypt; 3 Department of Pathology, Faculty of Medicine, Alexandria University, Alexandria, Egypt; Wadsworth Center, UNITED STATES OF AMERICA

## Abstract

Given the grave risk posed by intestinal cryptosporidiosis to vulnerable populations, the current research focused on a new therapeutic avenue. Olibanum (OL) extract and probiotic *Bacillus clausii* (*B. clausii*) and their combination were evaluated against *Cryptosporidium in vivo* in immunosuppressed mice via parasitological, ultrastructural, biochemical, and histopathological analysis. Liquid chromatography-mass spectrometry (LC–MS) analysis of OL revealed the predominance of incensole isomers in its chemical composition. Regarding oocyst count in stool, the highest percentage of oocyst reduction was achieved after combined therapy (98.4%) on the 19^th^ day post-infection, followed by *B. clausii-*treated group (87.6%), then the OL-treated group (87%). Similar ultrastructural alterations were noticed by scanning electron microscopy (SEM) following treatment by both OL and *B. clausii*, ranging from shrunken oocysts to severely deformed ones with extensive pits or protrusions. Nonetheless, their combination resulted in complete distortion of the oocysts, leading to their rupture. Biochemically, both OL and *B. clausii* treatment, either alone or in combination, provoked a decrease in the serum MDA levels, together with a corresponding rise in the mean levels of GSH. Concerning the histopathological findings, the best results were achieved following the combined OL and *B. clausii* treatment, which has restored villus architecture and remarkably ameliorated detrimental intestinal inflammation and goblet cell count. Finally, immunohistochemical analysis of the lymphocytic population of the intestinal villi revealed a statistically significant rise in the CD4+ /CD8+ ratio after treatment with either OL, *B. clausii* or their combination, which signals their proficient use for this therapeutic endeavour.

## Author summary

Cryptosporidiosis is a cosmopolitan food, and waterborne infection caused mainly by the obligate apicomplexan parasite, *Cryptosporidium parvum* (*C. parvum*). Cryptosporidiosis is responsible for debilitating diarrhea outbreaks, especially in children. Additionally, it is a major opportunistic infection that may lead to devastating chronic disease specially among immunocompromised patients. Current therapy of cryptosporidiosis depends on nitazoxanide (NTZ), the only FDA-approved medication for treatment of cryptosporidiosis. However, treatment failure of NTZ, especially in immunocompromised individuals, has been widely reported. Therefore, there is an urgent need to develop high efficiency and low-toxicity therapies against *Cryptosporidium*. Recently, phytotherapy and probiotics have unprecedentedly revolutionized the medical research, owing to their well-characterized pharmacological and safety profiles. Conspicuously, the present study presents significant findings on the promising therapeutic efficacy of Olibanum (OL) extract and probiotic *Bacillus clausii* (*B. clausii*) and their combinationin on cryptosporidiosis in immunosuppressed murine model. This efficacy was supported by significant reduction in the parasite burden, improved intestinal histopathological changes, obvious ultrastructural alternations in oocysts with significant antioxidant, immunomodulatory activities.

## Introduction

*Cryptosporidium* is among the principal causes of fatal diarrhea, particularly in children under five as it ranks the third most common enteric pathogen and accounts for 9% of childhood mortality globally [1]. In addition, it constitutes an important health hazard with an approximated loss of 12.9 million disability-adjusted life years (DALYs) and more than 8 million cases of food-borne illness annually [2,3]. As a known opportunistic pathogen, *Cryptosporidium* is responsible for chronic debilitating diarrhea in those with a compromised immune system, such as patients with acquired immunodeficiency syndrome (AIDS) and transplant recipients [4]. Furthermore, it threatens the livestock industry by affecting cattle, goats, sheep, rabbits, poultry, and pigs, leading to economic losses [5].

*Cryptosporidium* has a monoxenous complex life cycle with alternation between asexual and sexual reproduction in a single host [6]. Interestingly, there are forty-four species in the genus *Cryptosporidium* [7], of them, *Cryptosporidium parvum* (*C. parvum)* and *C. hominis*, usually infect humans. However, there are less common species that can cause severe disease such as *C. mortiferum*, *C. cuniculus*, *C. meleagridis, C. ubiquitum*, *C. canis,* and *C. felis* [8]. Notably, It is believed that *C. parvum* is the causative agent in the majority of human zoonotic illnesses [9]. The infective stage of the parasite is the oocyst, which is mainly transmitted via the faecal–oral route. Oocyst is 4–6 μm in diameter with a rigid, waxy wall made up of lipids and glycoproteins, which makes it resistant to extreme temperature (from −22 °C to 60 °C) and to chlorine, leading to difficult control in water supplies, swimming pools and livestock farms [2,10,11].

The only Food and Drug Administration (FDA)-licensed medication for treatment of cryptosporidiosis, nitazoxanide (NTZ), is a thiazole drug with broad-spectrum antiparasitic activity [12]. It acts predominantly through inhibition of pyruvate:ferredoxin oxidoreductase (PFOR), thus interfering with anaerobic energy transfer reactions leading to parasite growth restriction [13,14]. However, the main drawback of NTZ encompasses treatment failure in immunocompromised individuals, especially in HIV-infected children under 5, even with prolonged treatment [15]. Although over 200 drugs have been investigated against cryptosporidiosis, no ideal therapeutic regimen has been established yet [16]. Unfortunately, no vaccine is available for humans, though a vaccine for cattle has been approved recently [17].

Herbal-based products constitute efficient therapeutic alternatives due to their broad therapeutic potential, together with their environmental compatibility, availability, and low toxicity [18,19]. Undoubtfully, they possess a variety of bioactivities and offer novel chemical constituents that may yield previously undiscovered anti-cryptosporidial hits [20]. Additionally, in contrast to synthetic medications, they are easier to get approved for use in animal feed [21]. Olibanum (OL), also known as frankincense, is a natural oleo-gum resin that is extracted from *Boswellia* species [22]. It is the most well-known of the ancient plant resins that the ancient Egyptians utilized in embalming, as an incense and as a component of medicines and cosmetics [23]. Conspicuously, it is still successfully employed to treat various conditions, including gastric, hepatic, dermatological diseases and rheumatoid arthritis [22]. It showed prominent efficacy against diverse microbial pathogens such as *Staphylococcus aureus*, *Proteus vulgaris*, *Escherichia coli*, and *Candida albicans* [24]. Moreover, it exhibited *in vivo* antiparasitic activities against *Giardia lamblia* [25] and *Trichinella spiralis* [26] as well as *in vitro* activity against *Plasmodium falciparum* [27].

Probiotics are living microorganisms that offer tremendous health benefits to the host when administered in adequate amounts. They primarily exert their influence via regulating the gut microbiota, preserving and mending the intestinal barrier, enhancing nutrient absorption and metabolism, and boosting the immune system [28,29]. Among the spore-forming probiotic bacteria of the genus *Bacillus*, gram-positive, spore-forming, rod-shaped, and motile probiotic bacteria called *Bacillus clausii (B.clausii)* stand out with four strains (O/C, N/R, T and SIN) commercially available in the market. Multiple clinical investigations endorsed its effectiveness in treating gut microbiota dysbiosis and reducing antibiotic-associated diarrhea [30,31]. Concerning the anti-parasitic activity, a reliable study has declared its prominent anti-*Schistosoma mansoni* activity [32].

A combination of phytotherapy and probiotics may help mitigate the impact of this parasitic infection on the public health and livestock industry by addressing the gaps in treatment. Hence, the present study aimed to evaluate anti-*Cryptosporidium* activity of OL extract and *B.clausii* via parasitological, ultrastructural, biochemical and histopathological studies in an immunosuppressed mouse model. In addition, LC–MS analysis of OL to assess its chemical composition was also performed.

## Materials and methods

### 1. Ethics statement

All animal experimental procedures were conducted in accordance with the guidelines of the Ethics Committee for the experimental use of animals at the Faculty of Medicine, Alexandria University, Egypt under a study protocol approval number: 0306737.

### 2. Preparation and analysis of olibanum extract

Olibanum gum was procured as white solid clumps from the local Egyptian market. Initially, 50 gm of finely ground olibanum powder was dissolved in 200 mL of 70% ethyl alcohol for 48 hours. Subsequently, the combined olibanum/ethanol solution was centrifuged. Eventually, the supernatant was evaporated using a rotator evaporator, resulting in the formation of a sticky crude extract, which was dissolved in dimethyl sulfoxide [33,34]. Liquid chromatography-mass spectrometry (LC-MS) analysis was conducted on the prepared olibanum extract to identify and measure the concentrations of the various components of the extract.

### 3. Animals

Laboratory-bred Swiss albino mice (six weeks old, weighing about 22 ± 2 g) were acquired from the animal house of the Medical Parasitology Department, Faculty of Medicine, Alexandria University, Egypt. Male mice were utilized to minimize sex-related biological variabilities and avoid the potential confounding effects linked to the female estrous cycle, thereby improving experimental consistency. The mice were domesticated in a standard temperature (25 ± 2°C) and humidity (50 ± 5%) and had free access to food and drinking water. Mice stools were parasitologically examined for exclusion of any parasitic infection.

### 4. Parasite and mice infection

*Cryptosporidium parvum* oocysts were obtained from the Theodore Bilharz Research Institute, Giza, Egypt. Maintenance of *Cryptosporidium* oocysts was achieved by serial oral passage in Swiss albino mice every 2 weeks [35]. Isolation and concentration of the oocysts were performed through filtration and 10 min-centrifugation at 2500 rpm [36]. Afterwards, the purified oocysts were counted using a Neubauer haemocytometer and inoculated orally at a dose of 10^4^ oocysts/100 μl PBS/mouse for induction of cryptosporidiosis [35,37].

### 5. Immunosuppression and treatment

Cyclophosphamide was administered in four intraperitoneal doses of 70 mg/kg each, with one-week interlude to induce and maintain immunosuppression in mice [38]. Nitazoxanide (NTZ) was given to mice orally at a dose of 10 mg/kg once daily [39]. The olibanum extract was administered through oral gavage to mice in a single daily dose of 50 mg/kg [26]. Whereas *B. clausii* spores were isolated via centrifugation at 4000 g for 30 min, and then resuspended in sterile saline solution to obtain a final oral dose of one billion spores/mouse/day [32].

### 6. Animal grouping and experimental design

Thirty-six immunosuppressed male mice were allocated randomly in six equal groups, six mice each [35,40,41] as follows: Group I, non-infected non-treated mice; Group II, infected non-treated mice; Group III, infected nitazoxanide (NTZ) treated mice; Group IV, infected olibanum (OL) treated mice; Group V, infected *B. clausii* treated mice; Group VI, infected mice treated with both OL and *B. clausii*. Mice of groups II, III, IV, V and VI were infected with *C. parvum* oocysts two days after the second dose of cyclophosphamide. Treatment was initiated on the 5^th^ day post infection (PI) and continued for 14 consecutive days [39]. Mice of all studied groups were sacrificed on the 19^th^ day PI in order to assess the anti-*Cryptosporidium* activity of the tested drugs.

### 7. Evaluation of the treatment efficacy

#### 7.1. Parasitological analysis.

Stool samples were collected individually from each infected mouse on the 5^th^, before starting the treatment to confirm mice infection, then on the 12^th^ and 19^th^ day PI to monitor the effect of the tested drugs on oocyst burden after one and two weeks of treatment, respectively. The *Cryptosporidium* oocyst burden was counted in twenty high power fields of 50 μl MZN-stained smears of each infected mouse, and the mean count of oocysts in each mouse was calculated. Consequently, the mean count of oocysts in every infected group was calculated [42]. The percentage reduction (%R) in the *Cryptosporidium* oocyst count was calculated in according to the following equation:


$$\begin{array}{c} \text{Percentage reduction }(\%\text{R})=\frac{\text{N}-\text{n}}{\text{N}}\;\times \text{100} \end{array}$$

N: Mean oocysts count detected in the infected non treated control group

n: Mean oocysts count detected in each infected treated group

#### 7.2. Ultrastructural analysis.

Fresh stool pellets from mice in the different infected groups were collected on the 19^th^ day PI in 2.5% buffered glutaraldehyde phosphate and processed for examination of the ultrastructure of *Cryptosporidium* oocyst by scanning electron microscopy (SEM) [37,43].

#### 7.3. Biochemical analysis.

Serum malondialdehyde (MDA) and reduced glutathione (GSH) levels were assayed colorimetrically in mice from all studied groups on the 19^th^ day PI using a commercial kit according to the manufacturer’s guidelines.

#### 7.4. Histopathological analysis.

Ileal sections were excised from infected mice immediately after sacrifice and fixed in 10% formalin. After 24–48 hours, fixed segments were longitudinally cut and processed into paraffin blocks. Five-micron-thick sections were cut using a microtome to be mounted on glass slides and stained with hematoxylin and eosin (H&E) [35]. In the image analysis unit, Pathology Department, Faculty of edicine, Alexandria University, multiple photos at x100 power were captured using a microscope adopted camera. Photos were then evaluated using image J software to measure the villus height in at least 10 consecutive villi. The number of inflammatory cells per villus was counted at x100 power. Furthermore, goblet cell count was determined in Periodic Acid-Schiff stained (PAS) sections at low power (x200).

#### 7.5. Immunohistochemical analysis.

Serial five microns thick ileal sections were mounted on positively charged slides. They were stained by anti-CD4 (#IR649, clone 4B12, monoclonal mouse antibodies, Agilent, Dako) and anti-CD8 (#IR623, clone C8/144B, monoclonal mouse antibodies, Agilent, Dako). They were stained by DAB immunohistochemical technique using DAKO link 48 auto stainer. Multiple X400 photos were taken for at least 10 affected villi/mouse. Using image analysis software, CD4+ and CD8+ lymphocytes were counted/HPF, then the ratio was calculated [35].

### 8. Statistical analysis

All numerical data were analyzed with IBM SPSS software package version 20.0. (Armonk, NY: IBM Corp). The normality of continuous data was tested by the Shapiro-Wilk test. Quantitative data were expressed as minimum, maximum, median, and mean ± standard deviation. Statistical differences between the six studied groups were expressed using One way ANOVA test in conjunction with Post Hoc test (Tukey) for pairwise comparison between each two groups. Significant levels of the gained results were considered when P value < 0.05.

## Results

### 1. Liquid chromatography-mass spectrometry (LC–MS) analysis

LC-MS analysis of olibanum extract was performed, and the range of retention times was from 0.000 min. to 37.971 min. The main components were sixteen identified compounds which are listed in Table 1 according to their retention times and percentage composition. Incensole isomer (50.849%), followed by Incensole isomer (16.006%), Octyl Acetate (6.029%), Di(2-propylpentyl) Phthalate (4.448%), Incensol oxide (3.653%), Cembrenol (3.507%), 1-Octanol (2.798%), (Z)-1,2-dichloroethylene (2.605%), Bicyclo[9.3.1]pentadeca-4,14-diene, 4,14,15,15-tetramethyl-8-methylene-, (4E,11S)- (2.294%), Chloroform (1.95%), Ethyl palmitate (1.115%), Cembrene A (0.955%), β-Nerolidol (0.902%), 1-Dodecanone, 1-(4-methyl-5-tridecyl-2-thienyl)- (0.833%), 7,8-Epoxylanostan-11-ol, 3-acetoxy- (0.773%), 2-chloro-2-methyl-Butane (0.678%) and Ethyl laurate (0.606%) were detected.

**Table 1:** LC–MS analysis of OL extract.

Component	Ret. time(min)	Area(mV*s)	Area(%)	Height(mV)
(Z)-1,2-dichloroethylene	2.125	7216575.342	2.605	6383406.16
2-chloro-2-methyl-Butane	3.053	1877583.779	0.678	1667238.04
1-Octanol	12.575	7751274.939	2.798	6011091.88
Octyl Acetate	14.829	16701457.52	6.029	12511170.4
β-Nerolidol	19.58	2499730.643	0.902	1728291.28
Ethyl laurate	19.914	1679409.761	0.606	1346081.48
Cembrene A	24.02	2644552.828	0.955	1946371.92
Ethyl palmitate	24.167	3089335.62	1.115	1982854.32
Bicyclo[9.3.1]pentadeca-4,14-diene, 4,14,15,15-tetramethyl-8-methylene-, (4E,11S)-	24.64	6355097.973	2.294	4127837.17
Cembrenol	25.733	9714587.467	3.507	5952690.98
Incensole isomer	25.841	140863162	50.849	71412586.8
Incensole isomer	25.984	44340210.16	16.006	27646268.2
Di(2-propylpentyl) Phthalate	26.661	12320662.33	4.448	3230340.51
Incensol oxide	26.887	10118864.55	3.653	3822071.25
1-Dodecanone, 1-(4-methyl-5-tridecyl-2-thienyl)-	26.962	2308390.625	0.833	1504019.89
7,8-Epoxylanostan-11-ol, 3-acetoxy-	27.364	2140024.672	0.773	1188067.18

### 2. Parasitological analysis

*C. parvum* oocysts were identified and counted in MZN -stained fecal smears of the infected mice (Fig 1). On the 5^th^ day PI, a statistically non-significant difference in the mean oocyst count was detected between all infected groups. After administration of treatment, a statistically significant reduction in the mean *Cryptosporidium* oocyst load was revealed in the four infected treated groups (III, IV, V and VI) compared to the infected, non-treated group II on the 12^th^ and 19^th^ days PI. On comparing between the different treated groups, the best results were shown in group VI (treated with both OL and *B. clausii*) which recorded the greatest statistically significant reduction in oocyst shedding starting on 12^th^ day PI till achieving the lowest mean oocyst count (0.03 ± 0.03) and the highest percentage of oocyst reduction (98.4%) at the end of the experiment on the 19^th^ day PI followed by *B. clausii* treated group (V) with a mean of 0.23 ± 0.05 (87.6%), then OL treated group (IV) with a mean of 0.24 ± 0.04 (87%) on the 19^th^ day PI with a statistically non-significant difference between the two groups. Whereas the least percentage of oocyst reduction (76.2%) was observed in NTZ-treated group (III) with a mean oocyst count of 0.44 ± 0.16 on the 19^th^ day PI (Table 2).

**Table 2:** *Cryptosporidium* oocyst count in feces of mice in the different infected groups at various studied durations.

Count of Oocyst in stool	Non treated(n = 6)	NTZ treated(n = 6)	OL treated(n = 6)	B. clausii treated(n = 6)	OL&B. clausii treated(n = 6)	F	p
**5**^**th**^ **day**							
Median (Min. – Max.)	0.58(0.45–0.75)	0.53(0.40–0.80)	0.58(0.30–0.70)	0.58(0.35–0.85)	0.55(0.40–0.90)	0.116	0.976
Mean ± SD.	0.58 ± 0.12	0.56 ± 0.15	0.55 ± 0.14	0.59 ± 0.17	0.60 ± 0.18		
**12**^**th**^ **day**							
Median (Min. – Max.)	0.88(0.75–1.20)	0.50(0.40–0.60)	0.30(0.25–0.40)	0.28(0.20–0.35)	0.10(0.05–0.15)	74.648^*^	<0.001^*^
Mean ± SD.	0.91 ± 0.16	0.50 ± 0.07	0.31 ± 0.06	0.27 ± 0.06	0.10 ± 0.03		
**%R**		**45.1%**	**65.9%**	**70.3%**	**89.0%**		
**p**_**1**_		<0.001^*^	<0.001^*^	<0.001^*^	<0.001^*^		
**Significance**		p_2_=0.007^*^,p_3_=0.001^*^,p_4_<0.001^*^,p_5_=0.921,p_6_=0.003^*^,p_7_=0.023^*^					
**19**^**th**^ **day**							
Median (Min. – Max.)	1.88(1.55–2.05)	0.40(0.30–0.70)	0.25(0.20–0.30)	0.23(0.15–0.30)	0.03 (0 – 0.05)	250.006^*^	<0.001^*^
Mean ± SD.	1.85 ± 0.19	0.44 ± 0.16	0.24 ± 0.04	0.23 ± 0.05	0.03 ± 0.03		
**%R**		**76.2%**	**87.0%**	**87.6%**	**98.4%**		
**p**_**1**_		<0.001^*^	<0.001^*^	<0.001^*^	<0.001^*^		
**Significance**		p_2_=0.041^*^,p_3_=0.023^*^,p_4_<0.001^*^,p_5_=0.999,p_6_=0.023^*^,p_7_=0.041^*^					

% R: Percentage of reduction of each infected treated group relative to infected non treated control group.

F: F for One way ANOVA test, used in comparison between more than two groups.

Post Hoc test (Tukey) is used in pairwise comparisons.

p: p value for comparing between the studied groups.

p_1_: p value for comparing between infected non treated and each other groups.

p_2_: p value for comparing between infected NTZ treated and infected OL treated groups.

p_3_: p value for comparing between infected NTZ treated and infected *B. clausii* treated groups.

p_4_: p value for comparing between infected NTZ treated and infected OL&*B. clausii* treated groups.

p_5_: p value for comparing between infected OL treated and infected *B. clausii* treated groups.

p_6_: p value for comparing between infected OL treated and infected OL&*B. clausii* treated groups.

p_7_: p value for comparing between infected *B. clausii* treated and infected OL&*B. clausii* treated groups.

*: Statistically significant at p ≤ 0.05.

**Fig 1:**
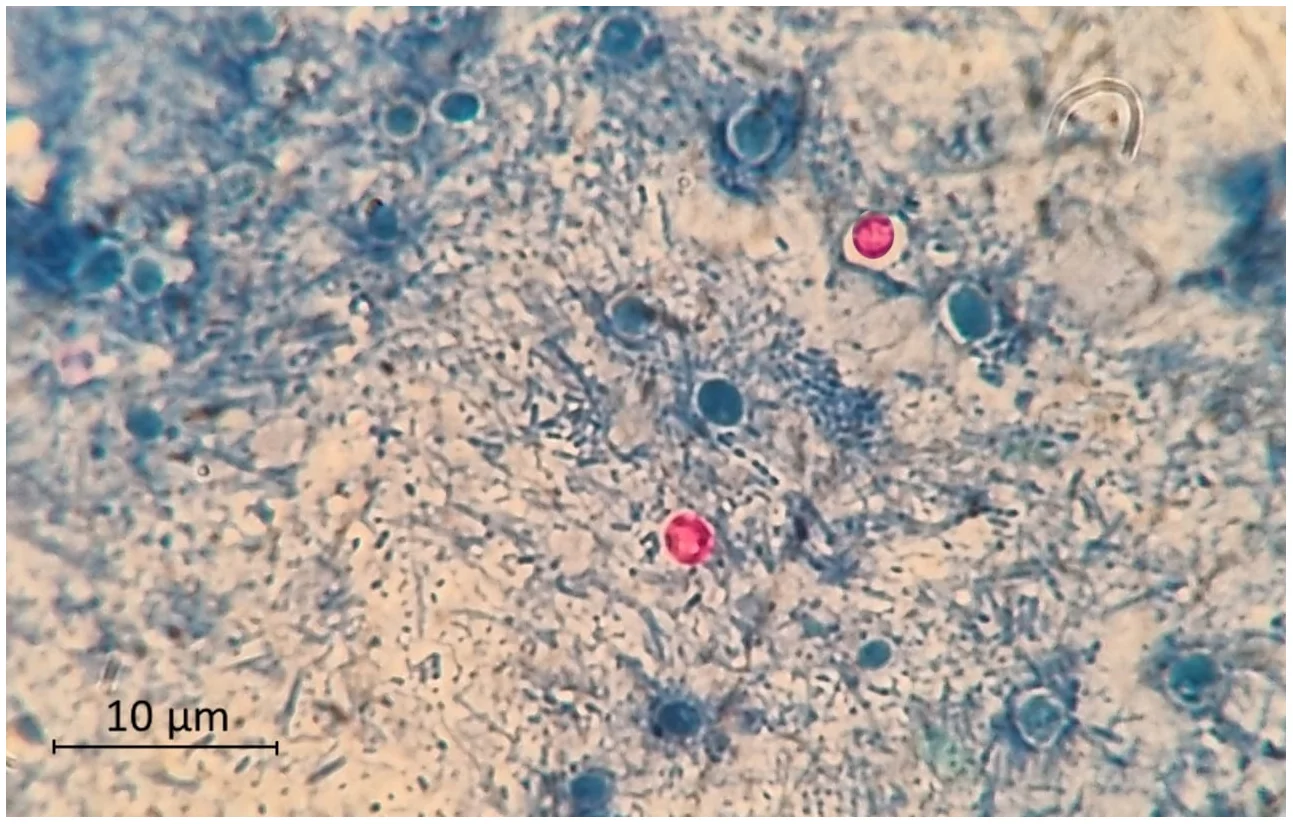
Light microscopy of *Cryptosporidium* oocysts in stool of infected mice-stained pink to red by MZN stain (x1000).

### 3. Ultrastructural analysis

SEM examination showed the non-treated *Cryptosporidium* oocysts with generally spherical shape and smooth, regular surface (Fig 2A). Meanwhile, oocysts retrieved from NTZ-treated mice revealed surface roughness with some compressions (Fig 2b). On the other hand, oocysts isolated from OL-treated mice demonstrated a variable range of morphological alterations. Most of the oocysts appeared shrunken (Fig 2C, 2D) with surface irregularities and multiple dimples (Fig 2C), while others exhibited extensive superficial pits and protrusions (Fig 2D). Some oocysts were deformed with numerous blebs and ridges (Fig 2E). In *B. clausii-*treated oocysts, various changes were observed including reduction in size (Fig 2F, 2G) with surface indentations and bullae (Fig 2F), numerous polyps (Fig 2G) or obvious distortion with spiky projections (Fig 2H). Whereas oocysts recovered from Group VI were completely irregular and distorted (Fig 2I). Noticeable oocyst mutilation and swelling with deep, wide depressions were also detected (Fig 2J). Several oocysts were ruptured with longitudinal cleavage and large surface blisters (Fig 2K).

**Fig 2:**
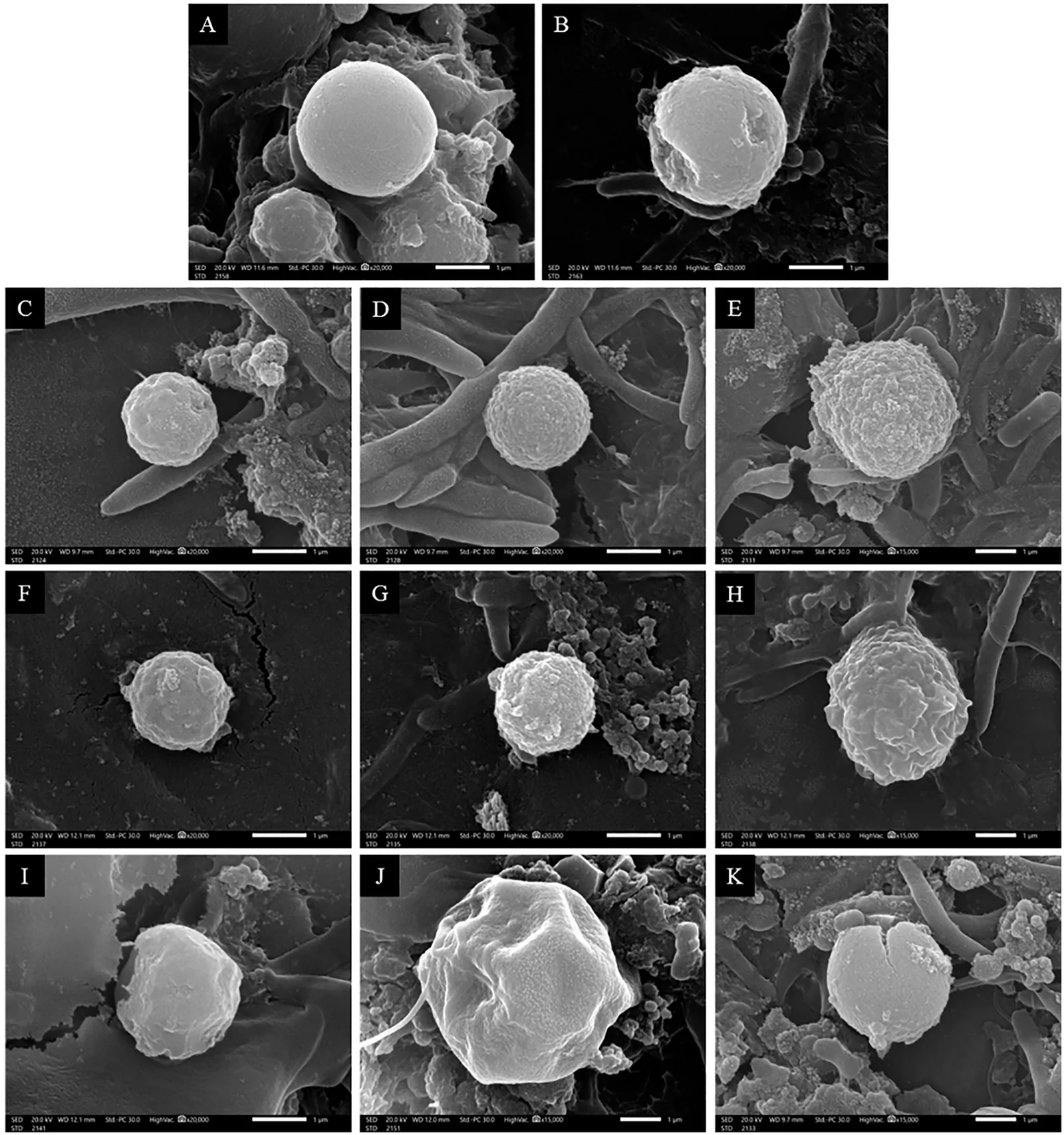
SEM of *Cryptosporidium* oocysts collected from stool of infected mice on the 19^th^ day PI. **(A)** Non treated oocysts viewing characteristic rounded shape with intact regular smooth body (x20,000); **(B)** NTZ treated oocysts appearing compressed with outer rough surface (x20,000); **(C–E)** OL-treated oocysts showing (C&D) Irregular shrunken body surface (x20,000); (C) Numerous dimples (x20,000); (D) Widespread pits and small protrusions (x20,000); **(E)** Distorted body with large papules (x15,000); **(F–H)** *B. clausii* treated oocysts illustrating (F&G) Reduced size (x20,000); (F) Indented irregular outer surface with multiple bullae (x20,000); (G) Apparently distended polyps (x20,000); **(H)** Prominent spikes (x15,000); **(I–K)** Combined OL & *B. clausii* treated oocysts demonstrating (I) Markedly disfigured body (x20,000); (J) Obviously swollen mutilated parasite with profound depressions (x15,000); (K) Ruptured organism with evident fissure throughout its diameter (x15,000).

### 4. Biochemical analysis

Experimental infection of mice with *C. parvum* induced a statistically significant increase in the serum levels of MDA in the infected groups (II, III, IV and V) when compared to the non-infected, non-treated control group (I). On the other hand, in response to treatment, serum MDA levels of the infected treated groups (III, IV and V) exhibited a statistically significant decrease compared to the infected, non-treated control group (II). Additionally, both infected OL and *B. clausii*-treated groups (IV and V) displayed a statistically significant decrease in the serum MDA levels compared to the infected NTZ-treated group (III). As to the infected treated group (VI), the combined OL and *B. clausii* treatment succeeded in reaching the most statistically significant decrease in MDA level among all infected treated groups. Interestingly, this remarkably low level of MDA accomplished in Group VI was statistically non-significant in comparison to the non-infected, non-treated group (I) (Table 3).

**Table 3:** Serum MDA and GSH levels of mice in the different studied groups on the 19^th^ day PI.

	Non infected non-treated(G I)	Infected					F (p)
**Non-treated(G II)**	**NTZ treated(G III)**	**OL treated(G IV)**	**B. clausii treated(G V)**	**OL & B. clausii treated(G VI)**
**MDA (nmol/ml)**							
Median (Min. – Max.)	6.27(5.73–6.78)	22.2(20.3–23.1)	16.2(15.6–18.4)	12.2(10.8–14)	11.31(9.85–14)	7.38(7.16–8.3)	F = 176.439p < 0.001^*^
Mean ± SD.	6.25 ± 0.47	22.10 ± 1.04	16.71 ± 1.04	12.21 ± 1.28	11.85 ± 1.67	7.59 ± 0.49	
**p**_**0**_		<0.001^*^	<0.001^*^	<0.001^*^	<0.001^*^	0.294	
**p**_**1**_			<0.001^*^	<0.001^*^	<0.001^*^	<0.001^*^	
**Significance**			p_2_<0.001^*^,p_3_<0.001^*^,p_4_<0.001^*^,p_5_=0.991,p_6_<0.001^*^,p_7_<0.001^*^				
**GSH (mg/dl)**							
Median (Min. – Max.)	2.88(2.42–3.55)	1.18(1.04–1.41)	2.27(1.48–2.57)	6.72(5.90–7.13)	4.73(3.92–5.73)	9.23(8.61–9.76)	F = 270.462p < 0.001^*^
Mean ± SD.	2.99 ± 0.44	1.21 ± 0.14	2.11 ± 0.42	6.64 ± 0.43	4.71 ± 0.62	9.20 ± 0.51	
**p**_**0**_		<0.001^*^	0.021^*^	<0.001^*^	<0.001^*^	<0.001^*^	
**p**_**1**_			0.018^*^	<0.001^*^	<0.001^*^	<0.001^*^	
**Significance**			p_2_ < 0.001^*^,p_3_ < 0.001^*^,p_4_ < 0.001^*^,p_5_ < 0.001^*^,p_6_ < 0.001^*^,p_7_ < 0.001^*^				

% R: Percentage of reduction of each infected treated group relative to infected non treated control group.

F: F for One way ANOVA test, used in comparison between more than two groups.

Post Hoc test (Tukey) is used in pairwise comparisons.

p: p value for comparing between the studied groups.

p_0_: p value for comparing between non infected non treated and each other groups.

p_1_: p value for comparing between infected non treated and each other groups.

p_2_: p value for comparing between infected NTZ treated and infected OL treated groups.

p_3_: p value for comparing between infected NTZ treated and infected *B. clausii* treated groups.

p_4_: p value for comparing between infected NTZ treated and infected OL&*B. clausii* treated groups.

p_5_: p value for comparing between infected OL treated and infected *B. clausii* treated groups.

p_6_: p value for comparing between infected OL treated and infected OL&*B. clausii* treated groups.

p_7_: p value for comparing between infected *B. clausii* treated and infected OL&*B. clausii* treated groups.

*: Statistically significant at p ≤ 0.05.

As compared to the non-infected, non-treated mice, the mean serum GSH level verified a statistically significant decline in the infected, non-treated mice and the infected NTZ-treated mice. However, treatment of the infected mice with OL, *B. clausii* or both provoked a statistically significant rise in the mean levels of GSH, whether compared to the non-infected, non-treated, the infected non-treated or the infected NTZ-treated mice. This rise in GSH level was higher in the OL-treated group than *B. clausii*-treated group with the highest level reported in the combined OL and *B. clausii*-treated group with a statistically significant difference between the three groups (Table 3).

### 5. Histopathological analysis

Results of histopathological examination are shown in Figs 3–4, and Table 4. Ileal sections from *C. parvum-*infected non-treated mice showed marked shortening and blunting of the villi with a mean villus height of 91.07 ± 15.60 μm. The villi in most areas were broad and edematous. Marked inflammatory infiltration (46.50 ± 4.23 Inflammatory cell /villous) was noted within their cores as well as in the lamina propria. It was formed mainly of lymphocytes, plasma cells and eosinophils. Additionally, goblet cell deletion was detected in the PAS-stained sections with a mean goblet cell count of 21.83 ± 3.54/ low power field (x200). On the other hand, all infected treated groups recorded a statistically significant improvement in the different studied histopathological parameters as compared to the infected non-treated group II. Interestingly, the highest statistically significant findings were noticed in group VI (the combined OL+ *B. clausii*, treated group), recording a mean villus height of 217.2 ± 6.28 μm, a mean inflammatory cell count of 10.67 ± 3.44/villous, and a mean goblet cell count of 61.83 ± 8.68/ low power field (x200). Subsequently, OL-treated group (IV) and *B. clausii-*treated group (V) achieved a nearly similar noticeable improvement in histopathological findings, which was statistically superior to NTZ-treated group III.

**Table 4:** Measurements of histopathological parameters in ileal sections of mice in the different infected groups on the 19^th^ day PI.

	Non treated(n = 6)	NTZ treated(n = 6)	OL treated(n = 6)	B. clausii treated(n = 6)	OL&B. clausii treated(n = 6)	F	p
**Villous height (microns)**							
Median (Min. – Max.)	97.07(66.22 – 106.1)	136.3(128.8 – 142.3)	168.3(158.2 – 178.2)	151.9(147.3 – 159.6)	218.1(206.8 – 223.5)	152.097^*^	<0.001^*^
Mean ± SD.	91.07 ± 15.60	135.7 ± 5.79	168.4 ± 8.38	152.8 ± 5.71	217.2 ± 6.28		
**p**_**1**_		<0.001^*^	<0.001^*^	<0.001^*^	<0.001^*^		
**Sig. bet. grps.**		p_2_<0.001^*^,p_3_=0.025^*^,p_4_<0.001^*^,p_5_=0.048^*^,p_6_<0.001^*^,p_7_<0.001^*^					
**Inflammatory cell count/villous**							
Median (Min. – Max.)	46.50(41.0 – 52.0)	26.50(23.0 – 31.0)	20.50(19.0 – 23.0)	20.0(18.0 – 25.0)	9.50(8.0 – 17.0)	104.470^*^	<0.001^*^
Mean ± SD.	46.50 ± 4.23	26.83 ± 2.99	20.67 ± 1.63	20.83 ± 3.06	10.67 ± 3.44		
**p**_**1**_		<0.001^*^	<0.001^*^	<0.001^*^	<0.001^*^		
**Sig. bet. grps.**		p_2_=0.020^*^,p_3_=0.024^*^,p_4_<0.001^*^,p_5_=1.000,p_6_<0.001^*^,p_7_<0.001^*^					
**PAS cell count/x200 field**							
Median (Min. – Max.)	21.0(18.0 – 27.0)	31.0(27.0 – 34.0)	44.0(39.0 – 49.0)	48.0(38.0 – 50.0)	63.50(49.0 – 71.0)	54.297^*^	<0.001^*^
Mean ± SD.	21.83 ± 3.54	30.67 ± 2.80	44.0 ± 3.74	46.17 ± 4.54	61.83 ± 8.68		
**p**_**1**_		0.044^*^	<0.001^*^	<0.001^*^	<0.001^*^		
**Sig. bet. grps.**		p_2_=0.001^*^,p_3_<0.001^*^,p_4_<0.001^*^,p_5_=0.946,p_6_<0.001^*^,p_7_<0.001^*^					

F: F for One way ANOVA test, used in comparison between more than two groups.

Post Hoc test (Tukey) is used in pairwise comparisons.

p: p value for comparing between the studied groups.

p_1_: p value for comparing between infected non treated and each other groups.

p_2_: p value for comparing between infected NTZ treated and infected OL treated groups.

p_3_: p value for comparing between infected NTZ treated and infected *B. clausii* treated groups.

p_4_: p value for comparing between infected NTZ treated and infected OL&*B. clausii* treated groups.

p_5_: p value for comparing between infected OL treated and infected *B. clausii* treated groups.

p_6_: p value for comparing between infected OL treated and infected OL&*B. clausii* treated groups.

p_7_: p value for comparing between infected *B. clausii* treated and infected OL&*B. clausii* treated groups.

*: Statistically significant at p ≤ 0.05.

**Fig 3:**
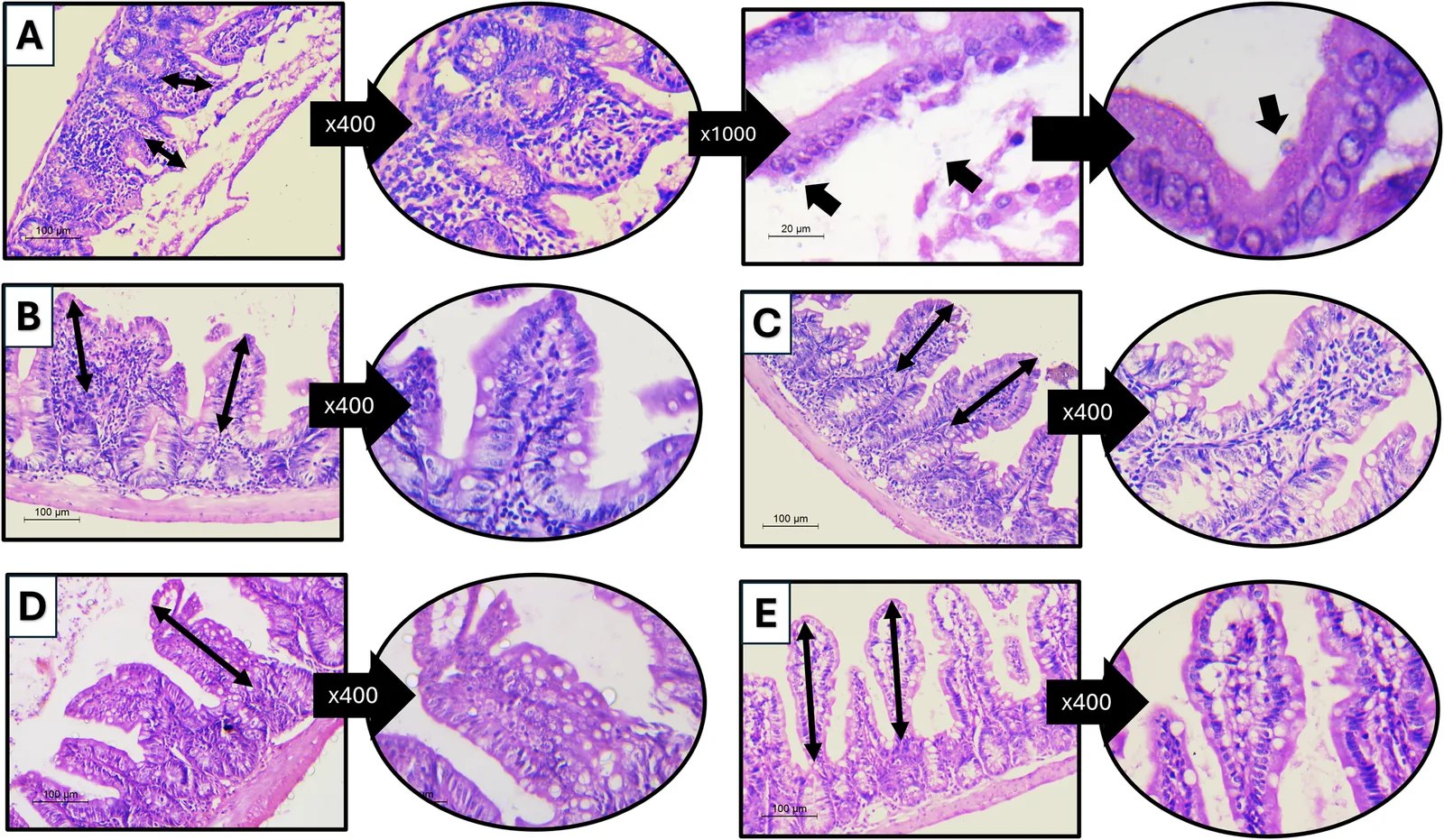
Histopathological study of H&E-stained ileal sections of infected mice on the 19^th^ day PI. Low power (x200) illustrates villous height (black lines) while x400 power shows villous inflammatory cell density. **(A)** Infected, non-treated mice showing evident blunting of the villi and shortening of villous height. The villi are broad and studded with numerous inflammatory cells. In X1000 power, *Cryptosporidium* oocysts are seen on brush border with inset highlighting the rounded oocyst with sporozoites inside; **(B)** NTZ treated mice revealing partial restoration of villous height and improved villous inflammation; **(C)** OL-treated mice and **(D)** *B. clausii* treated mice displaying evident improvement in villus architecture and height with prominent decrease in inflammatory infiltrate in the lamina propria; **(E)** Combined OL & *B. clausii* treated mice demonstrating marked improvement in both parameters. The villi restored their height and were slender and thin with minimal intravillous inflammatory infiltrate.

**Fig 4:**
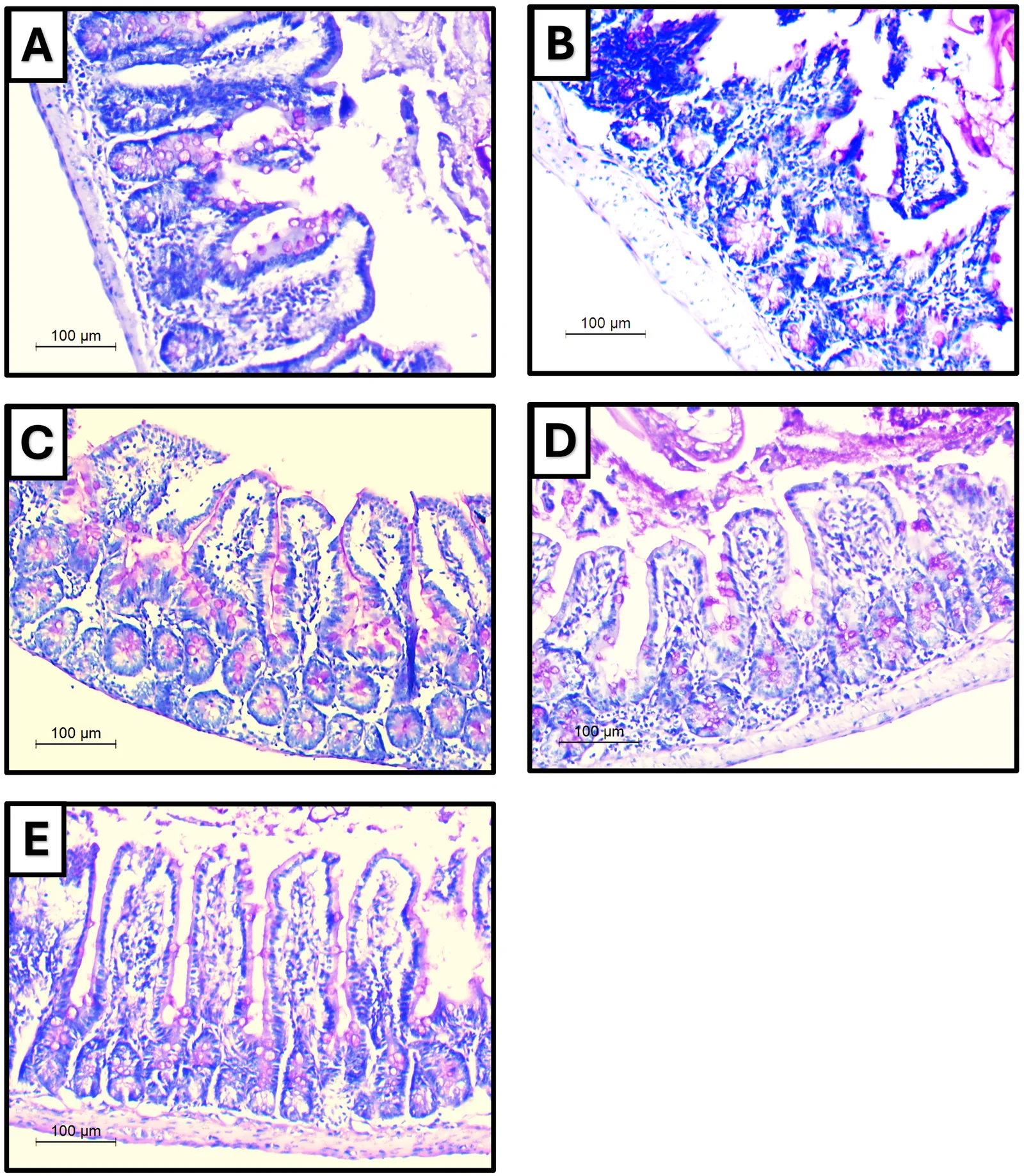
PAS-stained section of intestinal tissue of mice in infected groups (X200). Mucus cells are stained magenta red. (A) infected non treated group showing evident mucin depletion; **(B)** NTZ treated group presenting moderate increase in mucus cell count; **C)** OL-treated group and **(D)** *B. clausii* treated groups exhibiting more notable increase in mucus cells count; **(E)** Combined OL & *B. clausii* treated group illustrating obvious restoration of mucus cell population.

#### 7.6. Immunohistochemical analysis.

As regards the infected, non-treated group, the lymphocytic population of the intestinal villi showed predominant CD8+ T cells in comparison to CD4+ T cells, resulting in a low reversed CD4+ /CD8+ ratio (0.56 ± 0.16). Conspicuously, all infected-treated groups (III, IV, V and VI) revealed an increase in CD4+ T cells and a decline in CD8+ T cells, causing a statistically significant rise in CD4+ /CD8+ ratios (1.10 ± 0.10, 1.45 ± 0.26, 1.41 ± 0.12 and 1.79 ± 0.20, respectively) compared to the infected non-treated group II. The pairwise comparison between the four treated groups reported statistically significant differences in CD4+ /CD8+ ratio, except between groups IV and V (Fig 5 and Table 5).

**Table 5:** Intestinal CD4+/CD8+ Ratio of mice in the different infected groups on the 19^th^ day PI.

	Non treated(n = 6)	NTZ treated(n = 6)	OL treated(n = 6)	B. clausii treated(n = 6)	OL&B. clausii treated(n = 6)	F	p
**CD4+ /CD8+ Ratio**							
Median (Min. – Max.)	0.54(0.40 – 0.75)	1.09(1.0 – 1.21)	1.40(1.08 – 1.75)	1.46(1.22 – 1.50)	1.71(1.63 – 2.17)	40.457^*^	<0.001^*^
Mean ± SD.	0.56 ± 0.16	1.10 ± 0.10	1.45 ± 0.26	1.41 ± 0.12	1.79 ± 0.20		
**p**_**1**_		<0.001^*^	<0.001^*^	<0.001^*^	<0.001^*^		
**Sig. bet. grps.**		p_2_=0.017^*^,p_3_=0.042^*^,p_4_<0.001^*^,p_5_=0.995,p_6_=0.022^*^,p_7_=0.009^*^					

F: F for One way ANOVA test, used in comparison between more than two groups.

Post Hoc test (Tukey) is used in pairwise comparisons.

p: p value for comparing between the studied groups.

p_1_: p value for comparing between infected non treated and each other groups.

p_2_: p value for comparing between infected NTZ treated and infected OL treated groups.

p_3_: p value for comparing between infected NTZ treated and infected *B. clausii* treated groups.

p_4_: p value for comparing between infected NTZ treated and infected OL&*B. clausii* treated groups.

p_5_: p value for comparing between infected OL treated and infected *B. clausii* treated groups.

p_6_: p value for comparing between infected OL treated and infected OL&*B. clausii* treated groups.

p_7_: p value for comparing between infected *B. clausii* treated and infected OL&*B. clausii* treated groups.

*: Statistically significant at p ≤ 0.05.

**Fig 5:**
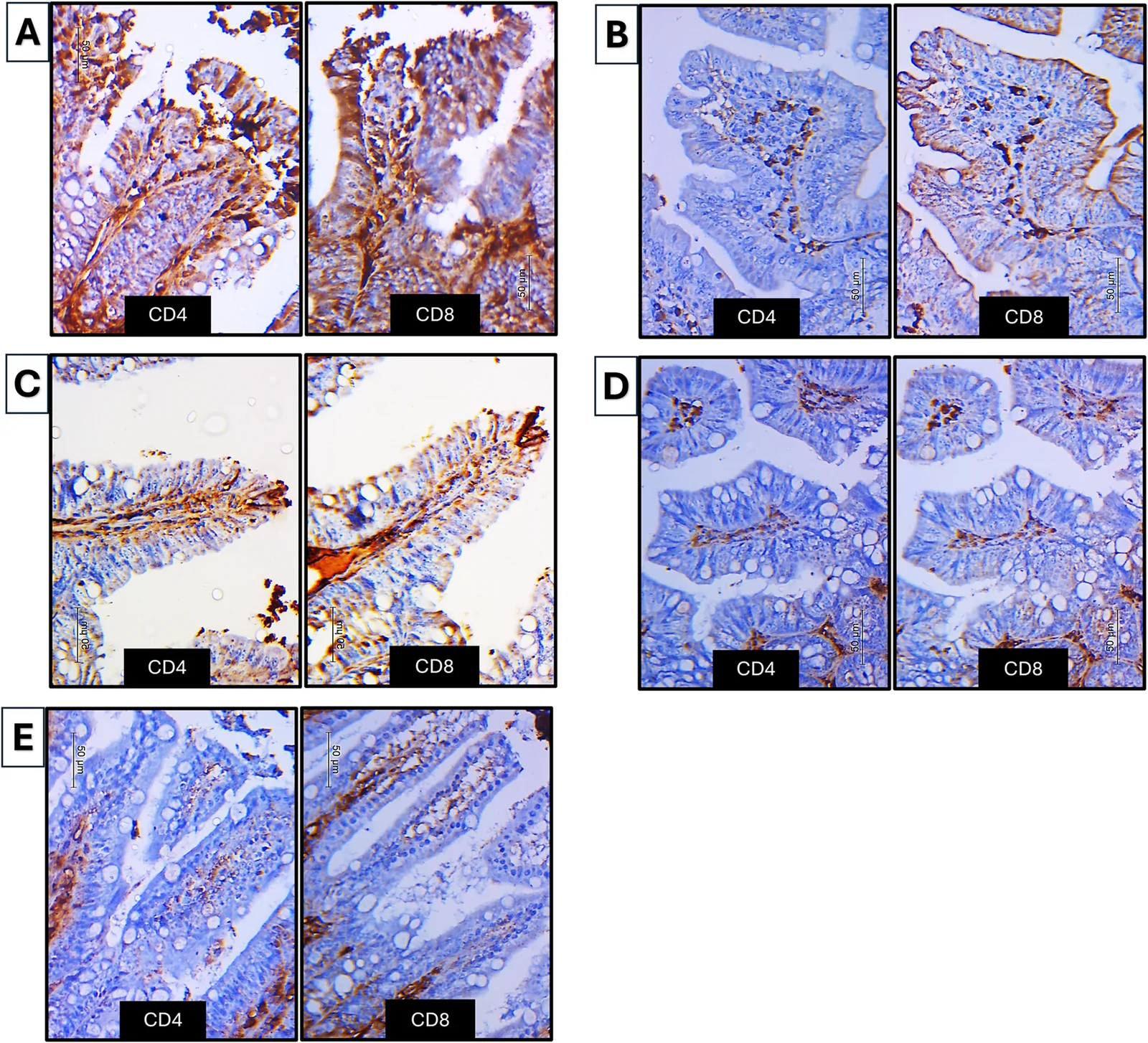
An immunohistochemical study of the intestinal tissue sections of infected mice (X400). The same field is stained using anti-CD4 and anti-CD8 monoclonal primary antibodies to show CD4+ and CD8+ lymphocytes. (A) infected non treated group showing increased CD8+ T cell population compared to CD4+ T cells (low CD4+/CD8+ ratio); **(B)** NTZ treated group displaying a considerable increase in CD4+/CD8+ ratio; **C)** OL-treated group and **(D)** *B. clausii* treated groups elucidating more prominent increase in CD4+/CD8+ ratio; **(E)** Combined OL & *B. clausii* treated group revealing the highest improvement of CD4+/CD8+ ratio.

## Discussion

Given that the target population for an efficient drug is predominantly constituted of young children, immunocompromised patients, and neonatal calves, crucial screening criteria for anti-*Cryptosporidium* medication include a safe and potent pharmacological profile with low probability of drug-drug interactions [44].

It is worth mentioning that the benefits of herbal medicine cannot be overlooked, with its use growing globally, as several organic compounds may be present in a single herbal extract, resulting in the synergistic effects of its constituents, which may contribute to its remarkable effectiveness [19]. Moreover, successful animal testing is necessary to confirm their safety and efficacy, either alone or in conjunction with other antiparasitic medications, to explore synergistic benefits of plant extracts against *Cryptosporidium*. In-depth research is constantly required to clarify the underlying molecular mechanisms behind the action of these plant extracts and their bioactive components [45,46].

Additionally, the tripartite relationship between intestinal parasites, the gut microbiota, and the host intestinal immune response has been previously explored to detect the critical impact of this relationship on human health. Pursuing research in this host–parasite–microbiota axis may aid in generating new therapeutics for intestinal parasites, minimizing their harmful influence on both public health and animal husbandry [47]. Preceding studies have shown that gut microbiota can affect parasitic clearance indirectly by modifying the host immune response and defences, in addition to their direct impact on specific parasites; therefore, probiotics and other microbial manipulation techniques might offer alternative or complementary approaches to treat intestinal parasite infections [47]. These reasons encouraged the authors to evaluate the anti-*Cryptosporidium* activity of OL extract and *B. clausii,* either alone or in combination, in an immunosuppressed mouse model.

In the present study, both OL and *B. clausii* achieved a significant drop in oocyst count with a considerable percentage of reduction, reaching 87% and 87.6%, respectively, at the end of the experiment. Moreover, similar morphological alterations were noticed by SEM following treatment, ranging from shrunken oocysts to severely deformed ones with extensive superficial pits or protrusions and blebs. Yet, their combination resulted in the highest percentage of oocyst reduction (98.4%) at the end of the experiment, hand in hand with complete distortion and noticeable oocyst mutilation and swelling, even rupture with visible longitudinal cleavage lines. As regards their antioxidant activity, both OL and *B. clausii* treatment, either alone or in combination, provoked a decrease in the serum MDA levels, together with a parallel rise in the mean levels of GSH.

The information gathered from previous studies suggested that OL has multiple modes of action, including anti-inflammatory and anti-leukotriene activities. It primarily reduces inflammation by inhibiting inflammatory molecules such as cyclooxygenase 2, 5-lipoxygenase, prostaglandin E, and inducible nitric oxide synthetase, which in turn prevents the production of leukotrienes [48]. It was reported that this anti-inflammatory activity reduced functional problems in the gut by improving motility, blocking intestinal smooth muscle contraction and preventing diarrhea without causing constipation [49]. To fully explore the relationship between of OL extract constituents and its activity in the current research, LC-MS analysis was performed. It revealed a predominance of incensole isomers and incensole oxide with other components such as octyl acetate, 1-octanol, cembrenol, cembrene A in the analysed sample, which is supported by previous analytic studies [50,51]. Incensole and its derivatives are cembrane-type diterpenes with promising anti-inflammatory activity ascribed to their inhibition of transcription factors (NF-κB and STAT3), which are implicated in the inflammation process [52].

Since probiotics are heterogeneous, diverse and strain-specific bacteria, several proposed mechanisms of action involving competition with enteric pathogens, modulation of the immune system and toxin receptors, and production of anti-microbial elements have been suggested [29]. It is worth mentioning that Lippolis et al. in 2013 declared expression variation of secreted proteins of *B. clausii* strains (O/C, N/R, T, and SIN). Each strain secreted a large profile of proteins into the extracellular environment directly in contact with the host compartments, thus mediating interactions with biological targets. Nonetheless, the authors proposed that the biological and pharmacological effects are ascribed to the cooperation of all metabolites [53]. As a result, *B. clausii* strains exhibited antimicrobial and immunomodulatory effects as well as expression alteration of genes involved in cell differentiation, inflammatory processes, and intestinal permeability [54,55].

In the current study, the best histopathological findings were detected after the combined OL and *B. clausii* treatment (group V), recording an improved villus height with a reduction of inflammatory cell count /villus, and an elevated mean goblet cell count. Intriguingly, the OL-treated group (IV) and *B. clausii-*treated group (V) achieved a nearly comparable improvement in histopathological findings. Previous studies have revealed an analogous improvement in the histopathological changes following treatment with OL in both giardiasis and intestinal trichinosis, which was substantiated by a significant decrease in the number of inflammatory cells, a low inflammatory score, and normal villous architecture at the affected level of the intestine [25,26]. Additionally, Al-Ghandour et al., in 2020, claimed that OL’s antioxidant activity contributed to the physiological maintenance of the enterocytes’ integrity and function [25]. Furthermore, in human enterocytes infected with Rotavirus, *B. clausii* spores and cell-free culture (CFS) supernatant prevented the production of reactive oxygen species (ROS) and the release of pro-inflammatory cytokines. They also restored cell proliferation, prompting a restart in the cell cycle progression and preventing apoptosis [56].

It is a well-known fact that the fundamental element affecting the probability and chronicity of cryptosporidiosis is the host's immunity, where immunosuppression, especially that affects T cell function, increases the severity of the disease [57]. Immunohistochemical analysis of the lymphocytic population of the intestinal villi, in the current study, exhibited a low reversed CD4+ /CD8+ T cell ratio in the infected non-treated immunosuppressed mice, which is in agreement with Fahmy et al., 2021 who reported similar results [35]. Whereas, all infected-treated groups, either OL, *B. clausii* or their combination, revealed an increase in CD4+ T cells and a decline in CD8+ T cells, causing a statistically significant rise in CD4+ /CD8+ ratio reverting back to a normal balanced ratio. Using *B. clausii* to treat children with allergic rhinitis, it showed anti-inflammatory and immunomodulatory effects on mucosal cytokines with the stimulation of T helper1 (Th1, CD4+ T cells) response, together with the inhibition of T helper2 (Th2, CD8+ cytotoxic T cells) hyperactivation [58]. On the other hand, the immunomodulatory impact of OL could be because it upregulates Th2 and their anti-inflammatory cytokines (IL-4, IL-10) and downregulates Th1 and their pro-inflammatory cytokines (IL-2, INF-γ, IL-6, and IL-12) [48,59] with a significant increase in the mean number of CD8+ T cells in the intestine following treatment with OL [26]. Though with seemingly contradictory mechanisms of action, both OL and *B. clausii* modified the immune reaction, each in its own way, resulting in a more balanced immune response as evidenced by the normalisation of CD4+ /CD8+ ratio, which is more noticeable in the combined group.

Finally, the superiority of OL and *B. clausii* and their combination over NTZ in all tested parameters might be explained by the fact that a healthy host immune system is necessary for NTZ’s effectiveness, since it has evident low efficacy in immunocompromised humans and animals. In fact, the innate immune response of host cells to infections has been proven to be amplified by nitazoxanide therapy with an increase in interferon activity [60]. At the earliest stages of infection, these innate responses are capable of restricting *Cryptosporidium* [61]; thus, NTZ would be ineffectual in immunocompromised hosts in case of an immune defect that prevents the host from producing an interferon-γ-dependent response [62].

## Conclusion

*Cryptosporidium* poses a substantial public health menace, predominantly in vulnerable populations, as well as a major livestock hazard. Due to the lack of effective treatment or a vaccine, there is a dire need to develop new therapeutic interventions. A combination of completely safe phytotherapy and probiotics offers a promising avenue for exploration. The current study demonstrated the superior activity of OL and *B. clausii* and their combination over NTZ in immunosuppressed mice model, regarding the parasitological, ultrastructural, biochemical, and histopathological findings. Notably, utilizing a single *Cryptosporidium* strain in the present study could not fully represent the diversity of the strains in natural infections, which may affect the relevance of the study findings. Future studies are suggested to assess the effect of purified components of OL such as incensole isomers and incensole oxide, alone or as hybrid molecules with other drugs, against other intestinal parasites and their underlying mechanisms in pre-clinical studies. Likewise, further research on *B. clausii* should be pursued, especially against various pathogenic parasites in immunocompromised patients.

## Supporting information

S1 File Liquid chromatography-mass spectrometry (LC-MS) analysis of olibanum extract. (PDF)
