# Supplementary material for: Unravelling the synergistic potential of Olibanum extract and Bacillus clausii in combating intestinal cryptosporidiosis in immunosuppressed mice
Source: PLoS Negl Trop Dis. 2026 Sep 30;20(9):e0014734. doi: 10.1371/journal.pntd.0014734 (PMC13626359; doi:10.1371/journal.pntd.0014734)

Analysis report

Sample Info

Analyst:	
Chromatogram:	№37 2024-05-23 14:00:12
Data file:	D:\Analytic 3\Projects\2091440 FID, TCD + MSD 2160084\.chromatograms\2024-05-23 14-00-12 0037 trial1 dr Hagar.chrx
Instrument:	Chromatec-Crystal 9000 S/N: 2091440 Firmware version: v 03.21.17.732
Method:	C:\ProgramData\Chromatec\Chromatec.Panel.UI\TempMethods\Trial 1_Scheduler_44d726741ca741d5b03e37974deecc10.mthx
Sample name:	trial1 dr Hagar
Sample volume:	1
Comments:	

Calculation result

Component	Ret. time	Area (mV*s)	Area (%)	Height (mV)	Detector
	2.125	7216575.342	2.605	6383406.16	MSD-1 : TIC
	2.541	5401865.26	1.95	4925174.7	MSD-1 : TIC
	3.053	1877583.779	0.678	1667238.04	MSD-1 : TIC
	12.575	7751274.939	2.798	6011091.88	MSD-1 : TIC
	14.829	16701457.52	6.029	12511170.4	MSD-1 : TIC
	19.58	2499730.643	0.902	1728291.28	MSD-1 : TIC
	19.914	1679409.761	0.606	1346081.48	MSD-1 : TIC
	24.02	2644552.828	0.955	1946371.92	MSD-1 : TIC
	24.167	3089335.62	1.115	1982854.32	MSD-1 : TIC
	24.64	6355097.973	2.294	4127837.17	MSD-1 : TIC
	25.733	9714587.467	3.507	5952690.98	MSD-1 : TIC
	25.841	140863162	50.849	71412586.8	MSD-1 : TIC
	25.984	44340210.16	16.006	27646268.2	MSD-1 : TIC
	26.661	12320662.33	4.448	3230340.51	MSD-1 : TIC
	26.887	10118864.55	3.653	3822071.25	MSD-1 : TIC
	26.962	2308390.625	0.833	1504019.89	MSD-1 : TIC
	27.364	2140024.672	0.773	1188067.18	MSD-1 : TIC
**Totals**		**277022785.5**	**100**	**157385562**	

Result of search

Ret.	Name	P, %	Match	R.Match	CAS#	NIST#
2.13	Dihydro-4,5-dichloro-2(3H)furanone	0.42	784	809	114434-99-0	288090
	Ethene, 1,1-dichloro-	4.73	870	895	75-35-4	114911
	Ethylene, 1,2-dichloro-, (E)-	23.71	922	939	156-60-5	290837
	1,2-Dichloroethylene	29.42	927	944	540-59-0	341803
	Ethylene, 1,2-dichloro-, (Z)-	41.67	937	954	156-59-2	291425
2.54	Acetyl chloride, dichloro-	0.04	666	669	79-36-7	230522

Result of search

Ret.	Name	P, %	Match	R.Match	CAS#	NIST#
2.54	Methane, dichloronitro-	0.47	748	775	7119-89-3	288259
	Methane, bromodichloro-	1.87	782	801	75-27-4	341923
	Methane, oxybis[dichloro-	9.98	839	866	20524-86-1	7702
	Trichloromethane	87.56	918	923	67-66-3	291570
3.05	1,6-Heptadien-4-ol, 4-propyl-	0.07	551	638	52939-61-4	114715
	Butane, 1-chloro-3-methyl-	0.27	584	608	107-84-6	227754
	2-Methyl-1,5-heptadien-4-ol	0.35	590	687	926-98-7	114800
	Pentane, 3-chloro-	4.18	682	727	616-20-6	58843
	Butane, 2-chloro-2-methyl-	94.42	808	858	594-36-5	227753
12.57	Octane, 3-chloro-	3.26	847	866	1117-79-9	114636
	Octane, 2-chloro-	4.15	853	868	628-61-5	114638
	Formic acid, octyl ester	6.05	864	873	112-32-3	109166
	Cyclopropane, pentyl-	24.1	899	920	2511-91-3	46309
	1-Octanol	25.08	900	909	111-87-5	228852
14.83	Lauryl acetate	1.27	799	799	112-66-3	351777
	Cyclopropane, pentyl-	2.62	818	885	2511-91-3	46309
	Acetic acid, decyl ester	2.97	821	821	112-17-4	374914
	Acetic acid, nonyl ester	5.77	839	839	143-13-5	232527
	Acetic acid, octyl ester	68.22	930	931	112-14-1	374911
19.58	Nerolidol 1	3.08	737	882	0--	285435
	Nerolidol 2	3.64	741	884	0--	285436
	Nerolidol	16.25	783	881	142-50-7	155682
	1,6,10-Dodecatrien-3-ol, 3,7,11-trimethyl-	19.23	787	903	7212-44-4	135322
	1,6,10-Dodecatrien-3-ol, 3,7,11-trimethyl-, (E)-	25.78	795	905	40716-66-3	108468
19.91	Ethyl tridecanoate	2.62	683	777	28267-29-0	107333
	Decanoic acid, ethyl ester	2.84	685	809	110-38-3	229107
	Undecanoic acid, ethyl ester	2.96	686	820	627-90-7	249094
	Pentadecanoic acid, ethyl ester	3.77	692	767	41114-00-5	233156
	Dodecanoic acid, ethyl ester	62.02	806	856	106-33-2	229100
24.02	1,3,6,10-Cyclotetradecatetraene, 3,7,11-trimethyl-14-(1-methylethyl)-, [S-(E,Z,E,E)]-	2.99	700	782	1898-13-1	42570
	(S,E)-8,12,15,15-Tetramethyl-4-methylenebicyclo[9.3.1]pentadeca-7,11-diene	4.63	713	805	386223-19-4	412767
	Bicyclo[9.3.1]pentadeca-3,7-dien-12-ol, 4,8,12,15,15-pentamethyl-, [1R-(1R*,3E,7E,11R*,12R*)]-	5.9	719	805	70000-19-0	42598
	(3E,7E,11E)-1-Isopropyl-4,8,12-trimethylcyclotetradeca-3,7,11-trienol	7.32	724	806	67921-02-2	412768
	(R,1E,5E,9E)-1,5,9-Trimethyl-12-(prop-1-en-2-yl)cyclotetradeca-1,5,9-triene	49.27	793	941	31570-39-5	384090
24.17	Heptadecanoic acid, ethyl ester	1.07	669	776	14010-23-2	232927
	Pentadecanoic acid, ethyl ester	1.56	680	808	41114-00-5	233156
	Nonadecanoic acid, ethyl ester	1.94	685	722	18281-04-4	233004
	Hexadecanoic acid, 2-methyl-, methyl ester	8.53	726	815	2490-53-1	68773

Result of search

Ret.	Name	P, %	Match	R.Match	CAS# NIST#
24.17	Hexadecanoic acid, ethyl ester	80.5	807	865	628-97-7 233204
24.64	Isopimara-9(11),15-diene	1.26	774	813	39702-28-8 413695
	Naphthalene, decahydro-1,1,4a-trimethyl-6-methylene-5-(3-methyl-2,4-pentadienyl)-, [4aS-	1.65	781	809	5957-33-5 13164
	1,3,6,10-Cyclotetradecatetraene, 3,7,11-trimethyl-14-(1-methylethyl)-, [S-(E,Z,E,E)]-	2.73	796	827	1898-13-1 42570
	Bicyclo[9.3.1]pentadeca-3,7-dien-12-ol, 4,8,12,15,15-pentamethyl-, [1R-(1R*,3E,7E,11R*,12R*)]-	20.52	870	905	70000-19-0 42598
	(S,E)-8,12,15,15-Tetramethyl-4-methylenebicyclo[9.3.1]pentadeca-7,11-diene	61.25	894	931	386223-19-4 412767
25.73	1,3,6,10-Cyclotetradecatetraene, 3,7,11-trimethyl-14-(1-methylethyl)-, [S-(E,Z,E,E)]-	1.11	751	799	1898-13-1 42570
	(S,E)-8,12,15,15-Tetramethyl-4-methylenebicyclo[9.3.1]pentadeca-7,11-diene	1.26	754	806	386223-19-4 412767
	Bicyclo[9.3.1]pentadeca-3,7-dien-12-ol, 4,8,12,15,15-pentamethyl-, [1R-(1R*,3E,7E,11R*,12R*)]-	4.13	779	831	70000-19-0 42598
	(R,1E,5E,9E)-1,5,9-Trimethyl-12-(prop-1-en-2-yl)cyclotetradeca-1,5,9-triene	14.86	809	915	31570-39-5 384090
	(3E,7E,11E)-1-Isopropyl-4,8,12-trimethylcyclotetradeca-3,7,11-trienol	66.24	851	889	67921-02-2 412768
25.84	1-Heptatriacotanol	0.41	681	731	105794-58-9 127968
	9-(3,3-Dimethyloxiran-2-yl)-2,7-dimethylnona-2,6-dien-1-ol	0.52	687	810	0-- 192156
	Formic acid, 3,7,11-trimethyl-1,6,10-dodecatrien-3-yl ester	0.86	702	767	0-- 132110
	(1S,2R,5E,9E,12R)-12-Isopropyl-1,5,9-trimethyl-15-oxabicyclo[10.2.1]pentadeca-5,9-dien-2-ol	11.6	802	858	22419-74-5 412799
	Isopropyl-1,5,9-trimethyl-15-oxabicyclo[10.2.1]pentadeca-5,9-dien-2-ol	81.52	873	904	0-- 412984
25.98	9-(3,3-Dimethyloxiran-2-yl)-2,7-dimethylnona-2,6-dien-1-ol	3.99	686	785	0-- 192156
	Oxirane, 2,2-dimethyl-3-(3,7,12,16,20-pentamethyl-3,7,11,15,19-heneicosapentaenyl)-, (all-E)-	5.22	693	750	7200-26-2 161611
	Thunbergol	8.33	707	800	25269-17-4 384002
	2,6,10-Dodecatrien-1-ol, 12-acetoxy-2,6,10-trimethyl-, (E,E,E)-	9.04	709	784	93787-91-8 164423
	Isopropyl-1,5,9-trimethyl-15-oxabicyclo[10.2.1]pentadeca-5,9-dien-2-ol	36.57	745	818	0-- 412984
26.66	Phthalic acid, di(oct-3-yl) ester	4.59	807	855	0-- 377723
	Phthalic acid, di(6-methylhept-2-yl) ester	5.43	811	860	0-- 377973
	Phthalic acid, 6-methylhept-2-yl octyl ester	6.43	815	866	0-- 377962
	Diisooctyl phthalate	16.27	837	883	131-20-4 62126
	Phthalic acid, di(2-propylpentyl) ester	35.72	857	909	0-- 377935
26.89	4,8,13-Cyclotetradecatriene-1,3-diol, 1,5,9-trimethyl-12-(1-methylethyl)-	2.25	657	730	7220-78-2 42995
	Ethyl iso-allocholate	3.48	670	685	0-- 43053

Result of search

Ret.	Name	P, %	Match	R.Match	CAS# NIST#
26.89	Tricyclo[20.8.0.0(7,16)]triacontane, 1(22),7(16)-	3.94	673	726	0-- 155907
	1-Heptatriacotanol	5.58	683	730	105794-58-9 127968
	1-Isopropyl-5,9,13-trimethyl-4,16-dioxatricyclo[11.2.1.03,5]hexadec-8-en-12-ol	54.78	765	878	21698-66-8 412985
26.96	1-Hexanone, 1-(4-methyl-5-tridecyl-2-thienyl)-	2.32	477	588	59782-61-5 68871
	Heptadecane, 9-hexyl-	2.95	483	545	55124-79-3 15194
	Tetrapentacontane, 1,54-dibromo-	11.53	517	583	0-- 156094
	Octadecane, 3-ethyl-5-(2-ethylbutyl)-	12.5	519	576	55282-12-7 16319
	1-Dodecanone, 1-(4-methyl-5-tridecyl-2-thienyl)-	14.8	523	615	59782-64-8 68876
27.36	1-Heptatriacotanol	2.29	557	671	105794-58-9 127968
	Cyclotriaconta-1,7,16,22-tetraone	2.84	562	628	0-- 155916
	Tricyclo[20.8.0.0(7,16)]triacontane, 1(22),7(16)-	2.84	562	690	0-- 155907
	Ethyl iso-allocholate	8.49	586	651	0-- 43053
	7,8-Epoxylanostan-11-ol, 3-acetoxy-	32.44	619	646	0-- 187609

Chromatogram

MSD-1 : TIC

Range from 0.000 min. to 37.971 min.

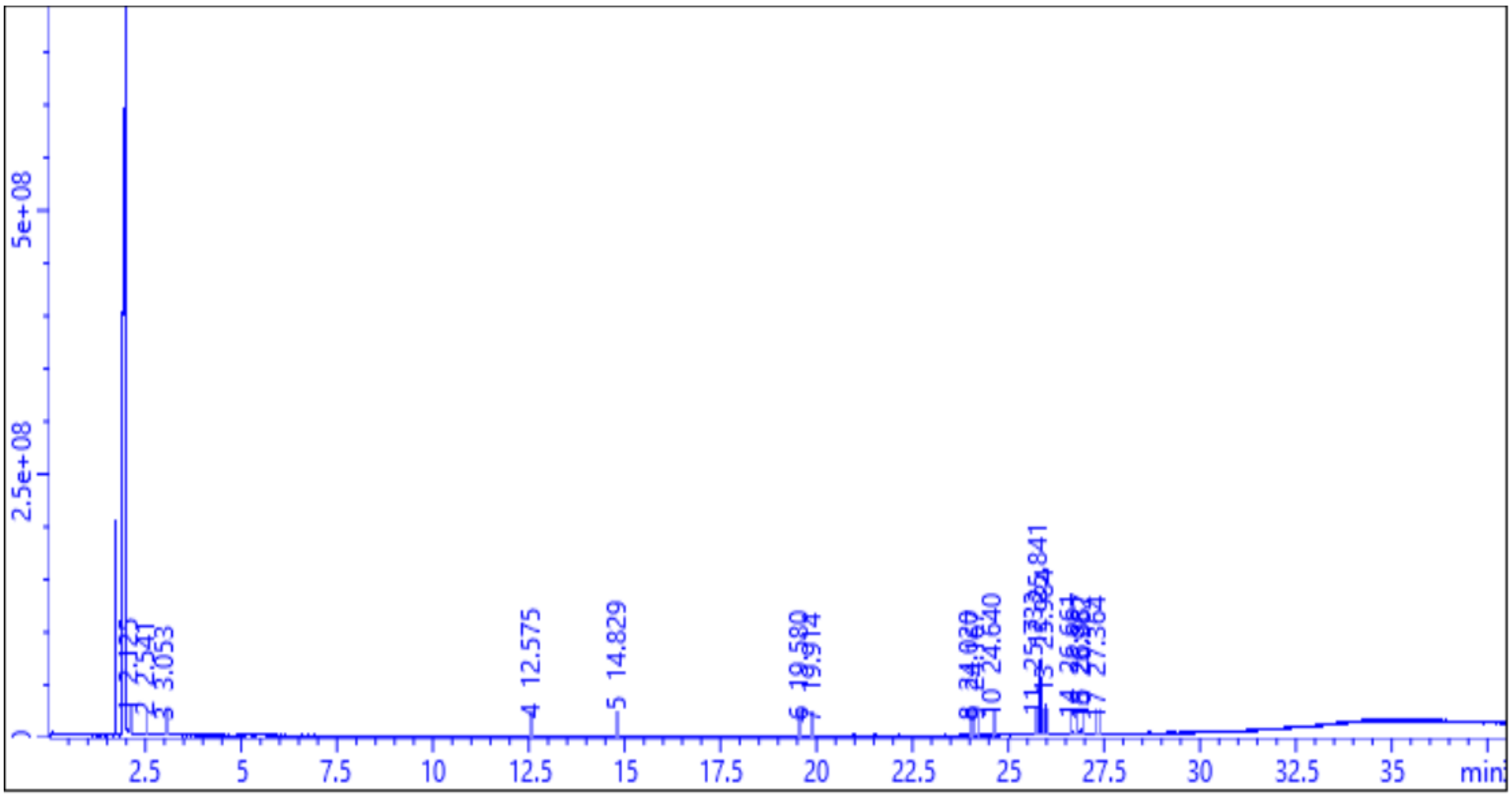

Supplement: Supplementary file 1 [file pntd.0014734.s001.pdf]
